# Addressing Pancreatic Cancer Disparities in Oregon’s Native American Population via Tribally Responsive Research Systems with The Confederated Tribes of Warm Springs

**DOI:** 10.21203/rs.3.rs-6637554/v1

**Published:** 2025-06-23

**Authors:** Jared Delaney, Lonnie James, Paige Farris, Kaitlin Greene, Brett Sheppard, Jackie Shannon, Jonathan Brody, Claymore Kills First

**Affiliations:** School of Medicine, Oregon Health & Science University; Knight Cancer Institute; Knight Cancer Institute; Knight Cancer Institute; Brenden-Colson Center for Pancreatic Care, School of Medicine, Oregon Health & Science University; Division of Oncological Sciences, Knight Cancer Institute, Oregon Health and Science University; Department of Cell, Developmental and Cancer Biology, Oregon Health & Science University; Division of Oncological Sciences, Knight Cancer Institute, Oregon Health and Science University

**Keywords:** Pancreatic Cancer, Indigenous, Native American, Cancer Disparities, Rural, Tribal Research

## Abstract

**Purpose:**

In Oregon, the incidence of Pancreatic Cancer is 2-times higher among American Indian and Alaska Native (AIAN) communities than among the rest of the population nationwide. We wanted to know if the previously validated Research in Oregon’s Communities Review System (ROCRS) could be adapted to investigate this disparity while upholding tribal sovereignty.

**Methods:**

We partnered with The Confederated Tribes of Warm Springs with the goal of adapting the ROCR System to address the pancreatic cancer disparity with a culturally responsive approach. One-on-one interviews with community members were conducted at the annual Pi-Ume-Sha Health Fair in 2023. Cancer-related data was requested from the Northwest Portland Area Indian Health Board. Barriers to healthcare access were identified and categorized using PESTLE analysis. A Tribal liaison combined this analysis with cancer-related data to create a cultural landscape. This was done in accordance with the ROCRS system.

**Results:**

This culturally responsive approach fosters trust and engagement in pancreatic cancer research and creates actionable insights for researchers while maintaining tribal sovereignty.

**Conclusion:**

The success of this model demonstrates the potential of tribally tailored research systems to improve participation and long-term collaborations with this underrepresented population.

## Introduction

Pancreatic cancer is soon to become the 2nd leading cause of cancer-related deaths in the United States [1]. In 2021, an estimated 100,669 people were living with pancreatic cancer in the United States; Pancreatic Ductal Adenocarcinoma (PDAC) accounts for approximately 90% of these diagnoses [2, 3]. In Oregon, the incidence of PDAC is 2-times higher among Native American (NA) communities than among the rest of the population nationwide [4]. In some rural medically underserved areas of Oregon, the incidence rate is as high as 16.8/100,000 individuals [5]. Additionally, Native American communities have the worst 5-year survival rate for PDAC (6.7%) among all US ethnic groups compared to the general population, with a 5-year survival rate of 11% [5].

When working alongside Native American (NA) populations, unique considerations must be taken because of their tribes having status as sovereign nations within the United States. Sovereign status is defined by the unique ability of tribes to self-govern. This status also provides the right of each tribe to organize and address healthcare needs in a personalized manner. Researchers should be aware of tribal sovereignty and each tribe’s ability to control all research within their population and borders if they wish to maintain a good relationship with the tribes they seek to investigate. The diversity in values of different tribes cannot be understated, as no two tribes are identical. With this as a foundation, each tribe must approve all research completed within their population and at the population or tribal community level. This typically fosters a different approach compared to working with other underrepresented minority groups in healthcare research, as tribal approval is needed from tribal governments and the communities they serve. Therefore, conducting research alongside American Indian and Alaska Natives (AIAN) represents a unique challenge for academic institutions.

AIAN communities mistrust research and healthcare due to the historical atrocities they have faced in the United States. The COVID-19 Pandemic has recently demonstrated how medical crises have placed many tribal nations in scenarios in which they have traded short-term public aid for long-term access to indigenous genomes [6]. This example demonstrates not only how mistrust can be born but how it can be difficult to return to these tribes and pursue research in the future. With this understanding our multi-disciplinary team, which includes clinicians, translational scientists, community outreach specialists, and population scientists, has established a collaboration with the Confederated Tribes of Warm Springs, Oregon’s largest tribal community. Overall, this team aims to increase cancer research in AIAN communities by increasing ownership, input, and development directly from tribes to research projects that are proposed to be conducted within their communities. We believe that this approach will ultimately provide a better quality of life for individuals in this tribe who are either at high risk of developing pancreatic cancer or living with this disease. We adapted the previously developed Research in Oregon Communities’ Review System (ROCRS) to the cultural needs of the Warm Springs tribal community [7]. ROCRS was developed in line with and by a rural community, ensuring a community-focused approach for reviewing incoming research. ROCRS was built on the idea that the engagement of community members will provide more access, cooperation, and interest in any research proposed to a community.

ROCRS is a systematic process for researchers to learn about a community and receive recommendations regarding the feasibility and realistic implementation of their study from an advisory of community stakeholders. Communities use this process to review, vet, and assess the requests of incoming researchers.

The town of Warm Springs is a rural community that sits on the Confederated Tribes of Warm Springs reservation with a population of approximately 3,395 people per US Census data. The Warm Springs reservation lies primarily within Wasco and Jefferson Counties and across smaller sections of six other counties: Clackamas, Marion, Gilliam, Sherman, Linn, and Hood River. According to data published by the Oregon Office of Rural Health, the Warm Springs service area had an unmet needs score of 22 for the year 2023 [8]. An unmet needs score was determined by nine overall variables divided into three categories: availability of providers, ability to afford care, and utilization of care. Provider availability was further defined by travel time to the nearest patient-centered primary care home, primary care capacity, dentists per 1,000 population, and mental health providers per 1,000 population. The ability to afford care is defined as the percentage of the population living between 138% and 200% of the federal poverty level. Finally, utilization was defined by inadequate prenatal care rate per 1,000 births, ambulatory care sensitive conditions/preventable hospitalizations per 1,000 population, non-traumatic dental visits to an emergency department per 1,000 population, and emergency department mental health/substance use visits per 1,000 population. Each variable was calculated from to 0–10 with the highest assignable score being 90.

The Warm Springs unmet needs score is the worst for 2023 as compared to the East Klamath service area, which had a score of 26, and the Swisshome/Triangle Lake service area, which had a score of 28 [8]. The other regions chosen for comparison round out the bottom three regions for the state of Oregon. The SW Portland service area received the highest score in 2023 of 78 [8]. Some barriers that contribute to this disparity include, but are not limited to, high rates of poverty, physical isolation, historical traumas to Native Americans (NA), and distrust in the health care system. The multi-disciplinary team at OHSU’s Knight Cancer Institute, specifically the Community Outreach and Engagement (COE) team and the Brenden-Colson Center for Pancreatic Care, aims to build trust with the Confederated Tribes of Warm Springs to accomplish shared goals.

The first step in the ROCR System is the development of a regional cultural landscape document. This document informs incoming research teams about important cultural norms, governmental interactions, relationships, and other factors that impact the feasibility and success of research implementation. Researchers are expected to review this document prior to submitting research proposals through the ROCRS system for review by the Warm Springs’ Health and Welfare Committee. Cultural landscapes include the presentation of data on cancer incidence and mortality, lifestyle, behavioral, and environmental data, as well as interviews with community members that provide the proper context for quantitative data. Although not all encompassing, cultural landscapes allow researchers to respond to and improve project viability prior to the presenting of the proposed project to the tribes.

## Methods

The methods mentioned in this paper do not consider human subject research, as deemed by OHSU IRB.

### One on One Interviews with Community Members

In accordance with Step 1 of establishing a culturally tailored ROCR System, we conducted anonymous one-on-one interviews with Warm Springs community members at the annual Pi-Ume-Sha health fair in 2023 (conducted by authors CKF, JRB, and JD). This health fair is part of the larger Pi-Ume-Sha treaty day celebration, commemorating the signing of the Treaty of 1855 between the NA of Middle Oregon and the United States, which established the Confederated Tribes of Warm Springs Reservation. The interviewers were trained by the Central Oregon-located COE team. During each interview, one team member documented the session, while the other spoke with a community member. Participating community members were reimbursed with twenty-dollar gift cards for use at a local coffee shop. A member of the team summarized the written documentation of the interviews thematically into six PESTLE categories: environmental, political, economic, social, legal, technological, and environmental.

**Figure 1:**
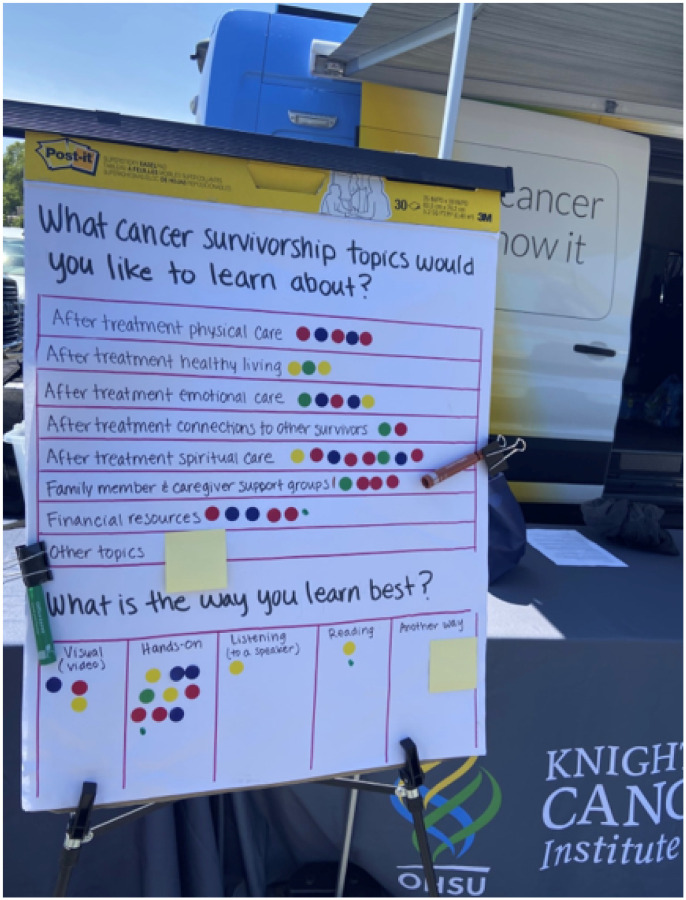
Cancer Survivorship Board with Sticker Responses a. Figure one demonstrates a picture of the doc survey board. It is a large white paper board with questions written out in English. The answer choices are listed below their respective questions. A sticker is placed in a box next to the answers, denoting a community members agreeance with the answer.

Community members also identified their most efficacious learning styles, what cancer survivorship topics they would like to learn about, and what kinds of cancer survivorship resources are needed during the health fair. We used a large board to list questions and asked community members to place a sticker on the topical answers they agreed with the most (see Fig. 1). Community members were not limited by the number of stickers that they could place.

#### Cultural Landscape Development

This community input was combined with the data on pancreatic cancer. Cancer-related data were requested by the Health and Welfare Committee of the Northwest Portland Area Indian Health Board and corroborated with the local Indian Health Service (IHS) agency and state data. ROCRS utilizes PESTLE analysis to develop questions for community members and helps the team organize the document’s presentation. During the initial phases of this study, a cultural liaison was hired. The cultural liaison spearheaded the development of the cultural landscape and helped to aggregate all cancer data.

The Warm Springs Health and Welfare Committee agreed to use the ROCR System to review the team’s multi-layered project. The Health and Welfare Committee made minor modifications to the forms and processes used as part of the review system to more accurately reflect the unique aspects of the Confederated Tribes of Warm Spring as a sovereign nation. One important modification is the maintained connection between the Health and Welfare Committee and a community advisory committee, which will also participate in reviewing incoming researcher requests. While the community advisory committee is yet to be formalized, having input from tribal members with more specific knowledge and life experience related to research topics in addition to the leadership perspective of members of the Health and Welfare Committee was identified as an important addition to the ROCR System in Warm Springs.

#### Project Approval and Future Project Goals

After a careful review of the project, the Health and Welfare Committee approved the team to move the proposed project components forward. The team is beginning the overall project by offering community education events in all parts of the Warm Springs community to talk more about the importance of pancreatic cancer in NA communities. One part of our project was the development of a NA PDAC tissue bank and registry. There is an overall group acknowledgement of how much more time a tissue bank will take to initiate, as tribal sovereignty related to data use agreements and storage of collected data and specimens is paramount. Cultural beliefs about tissue extraction and banking could possibly make the development of a proposed tissue bank and registry difficult. The methodological implementation of parts of our overall project is uniquely effective because of our close relationship with the Health and Welfare Committee, direct NA researcher involvement, leveraging a multi-disciplinary team, and using the ROCR System.

## Results

### Interviews conducted.

Ten one-on-one interviews were conducted and documented in total. Interview themes were drawn into separate categories, and the documented results will help develop a more robust, research-actionable cultural landscape. Politically, interviewees expressed a lack of trust between tribal members and the U.S. healthcare system. Provider consistency is also difficult for patients at Warm Springs because of high provider turnover and vacated positions at the clinic. Economically, the interviews expressed concerns over access to healthy food, citing that events outside of the reservation have healthy food options, while those on the reservation do not. Warm Springs also do not have a formal grocery store. Socially, members of the Warm Springs community enjoy events and seek community building opportunities, especially those that focus on health. The fitness center offers a space to engage in many of these activities but is currently limited to 8am to 5pm, making it difficult for those who are employed. Interviewees also expressed that many community members masked their battle with mental and physical health and were unwilling to seek help. Technological concerns center on transportation to and from reservations. This is difficult to achieve without the use of private vehicles. Warm Springs are over 640,000 acres, meaning that many people must travel for services, including healthcare and access to potable water, creating an obstacle for those without transportation. Legally, interviewees expressed confusion between IHS, HealthComp, Medicare, and private insurance regarding who covers what and how much they would cover. They also expressed that many people in Warm Springs seek primary care or second opinions outside of the reservation, but referrals for such activities are hard to obtain and that it can be difficult to cover transportation costs. Environmental concerns from interviewees included water contamination and clean water availability. The results are summarized and presented in Table 1.

#### Constructive dialogue about gaps and ways to address them

People want to be informed at every step, so they not only know what to do but also feel comfortable doing so. The closest cancer center is located at St. Charles Medical Center in Bend, which is 57 miles away. In terms of education, there are many services available at Warm Springs that previously did not exist (e.g., physical therapy, acupuncture, crisis services, free buses), but many people do not feel as if these services are advertised well enough. Community members seek information regarding health education with a desire for more available health information at community centers such as libraries, clinics, and local businesses. Tribal members have a strong desire for authority over their own health but feel that the information needed to be truly autonomous is difficult to attain.

#### Dot survey performed.

The results of the (dot survey) board questions are as follows: When asked “What cancer survivorship topics would you like to learn about: the most chosen answer was “Financial resources”. The second most selected answer was “Family member and caregiver support groups” while the third was “After treatment spiritual care”. When asked “What cancer survivorship, supportive resources are needed” the highest response was “Spiritual.” The second highest response was “Emotional” tied with “Mental.” When asked, “How do you learn the best?” Most respondents said “Hands-on”. The results are summarized in Tables 2–4. While Figure 1. Shows the exact way in which the data was collected.

#### Results from the quantitative data collection support obtained.

Both Jefferson and Wasco counties have higher proportions of American Indian or Alaskan Native residents. Jefferson County has the 4th highest population of American Indian or Alaskan Native residents in the state. In terms of pancreatic cancer incidence, Jefferson County reported the 5th highest incidence rate (14.5/100,000), whereas Wasco County reported the 4th highest incidence rate (15.5/100,000). Wasco County reported the highest overall cancer mortality rate (162.4 deaths/100,000 residents), indicating barriers to cancer care and poor outcomes. Jefferson County reported the 5th lowest rate of older adult men aged ≥ 65 years who are up-to-date on a core set of clinical preventive services (flu shot past year, PPV shot ever, and colorectal cancer screening), indicating barriers to care. Jefferson County reported the 5th highest rate of diabetes and 4th highest rate of obesity in the state, underscoring the lack of healthy food options.

#### Tribal liaison hired in Warm Springs.

With guidance from the Health and Welfare Committee, we advertised a tribal liaison position during the Pi-Ume-Sha Health Fair and identified an excellent candidate. The tribal liaison has multiple roles within the project, but ultimately serves as a bridge between the research team and the tribe to ensure that a truly collaborative and trusting relationship develops. It is essential that the tribal liaison is a member of the community with intimate cultural, social, and political knowledge of the tribe and its members. In addition to working closely with the researchers, the tribal liaison leads the development of the cultural landscape, attends the Health and Welfare Committee meetings, coordinates and plans culturally appropriate events in Warm Springs, and facilitates meetings with tribal stakeholders.

The importance of a tribal liaison cannot be overstated, as their lived experiences and direct involvement in the community provide researchers with the knowledge necessary to develop projects in a way that is beneficial to the community and will be accepted by tribal members. Another benefit of hiring a tribal member is their ability to secure a physical space within the clinic to work, providing direct access to clinic staff and the Health and Welfare Committee. Because of this proximity, we were able to move this project forward at a much faster rate than our original timeline. Ultimately, hiring a tribal liaison may be the most important step towards building a trusting and productive relationship between researchers and the tribe.

#### Initiation of two research projects.

The approval of two projects using the ROCR System through community feedback forms were completed. The first project involved community educational events designed for tribal members to openly discuss and learn about PDAC and cancer in general. An important component of these events is the opportunity for community members to provide direct feedback to researchers on project design and to provide historical and cultural insights into the current state of cancer care in their community. These events were designed by our tribal liaison and built around the traditional gatherings for meals that the tribe would host. The events were created to familiarize community members with the researchers in order to increase engagement and community building. The events were and are informed by our previously collected data on what patients want to learn and how they learn best. Each event begins with a meal, traditional prayer, and introductions. Researchers and community members eat together and visit prior to a larger circle of discussion about PDAC and cancer in general. Detailed notes were taken on community members’ experiences, views, concerns, and suggestions regarding cancer and cancer care. These notes were then used to further tailor future events to community needs.

Exploration of the development of a Native American PDAC tissue registry was the second project to be initiated. This tissue registry is the first NA PDAC tissue registry of its kind and serves as evidence to support our careful approach to trustful relationship building through the ROCRS framework.

## Discussion

Overall, the themes of the interviews revealed many aspects that may be important to researchers when designing their projects. One is the geographic size of the Warm Springs reservation, making it difficult to commute on the reservation and for researchers to engage with different individuals in this vast area. The other is how willing Warm Springs community members are to get involved with community events, especially those centered around health. There is a clear yearning for better health outcomes, which is hindered by availability, education, and trust. These are important aspects that can dictate the success of future research in this setting. The cultural landscape is undergoing further development by the community liaison to add further details around the IHS and its role within the community. The answers to the questions asked in the dot survey will inform the community of the projects we plan to introduce.

Cultural beliefs directly affect what community members want to see, as reflected in their resources. Spiritual, emotional, and educational initiatives can be tailored to improve user satisfaction. Financial support resources are also important for underserved populations with barriers to their care. One of our subtasks for the Kuni Foundation grant specifically addresses financial concerns and designates funding to cover treatment-related expenses. Additionally, in the subtask, we plan to establish a Patient Navigator program. Specifically, we will fund patient navigators who will be a useful resource to help NA patients overcome barriers to clinical trial participation by 1) providing culturally appropriate patient education; 2) disseminating patient education tools addressing commonly identified concerns among NA patients; 3) coordinating and financing travel, lodging, and meal expenses; 4) implementation of telehealth capabilities; 5) coordinating local administration of chemotherapy (when trial-appropriate); and 6) assisting with medical referrals to cancer treatment centers.

Learning styles will also impact community education events, further tailoring our efforts and increasing the efficacy of these educational events. When conducting research within Native American communities, more time and consideration in establishing trust and shared decision-making should be taken due to the historical traumas committed to them. Community leaders and members must be involved, and tribes must ensure their data autonomy. American Indian and Alaskan Native researchers can also improve community trust and help research teams filter ideas prior to presenting them to tribes. The groundwork demonstrated that ROCRS is a viable tool for use with American Indian and Alaskan Native communities. Obstacles to this work include clear communication and maintaining that our team is on the same page as the Health and Welfare Committee.

## Conclusion

Implementing an adapted ROCRS for Tribes to receive, review, and co-design incoming research requests is a unique approach that can increase their acceptance and participation in research and clinical trials. This novel approach is imperative not only because of the disparities seen within this community but also because of the high amounts of mistrust within this community. Future work will complement what has already been completed by bringing clinical trials through Warm Springs using this modified ROCR System, as well as the expansion into other communities and recreating this process at other cancer centers.

## Figures and Tables

**Table 1.** PESTLE Analysis of 1 on 1 Interviews

	*PESTLE Category*					
	Political	Economic	Social	Technology	Legal	Environment
*Analysis Summary*	Mistrust in the US healthcare system	Limited access to healthy foods	Importance of social events	Private vehicles are imperative for transportation	Confusion about insurance coverage	Concerns over water contamination
	High healthcare provider turnover		Spaces to hold events are limited		Referrals are hard to obtain	
			Mental and physical health battles are masked		Second opinions on medical issues are often desired	

a. The Table is broken into 6 categories: Political, Economic, Social, Technology, Legal and Environment. The statements in each category were chosen by analyzing themes from the one on one interviews.

**Table 2.** Dot survey question #1 with results

	First question on dot survey	
	*Q: What cancer survivorship supportive resources are needed?*	
		Number of responses
Categories listed on survey	Physical	10
	Spiritual	14*
	Emotional	13
	Mental	13
	Something Else	3

Note: Respondents were allowed to answer multiple times

* Highest number of responses

a. Table 2. is from the dot survey and shows the main question being asked “What cancer survivorship supportive resources are needed?”. Answers are listed with the numbers of stickers placed in their respective box,

**Table 3.** Dot survey question #2 with results

	Second question on dot survey	
	*Q: What cancer survivorship topics would you like to learn about?*	
		Number of responses
Categories listed on survey	After treatment physical care	6
	After treatment healthy living	3
	After treatment emotional care	6
	After treatment connection to other survivors	2
	After treatment spiritual care	8
	Family Member and caregiver support groups	9
	Financial Resources	10*
	Other topics	0

Note: Respondents were allowed to answer multiple times, “Other Topics” was a write in answer space

* Highest number of responses

a. Table 3. is from the dot survey and shows the main question being asked “What cancer survivorship topics would you like to learn about?”. Answers are listed with the numbers of stickers placed in their respective box,

**Table 4.** Dot survey question #3 with results

	Third question on dot survey	
	*Q: How do you learn best?*	
		Number of responses
Categories listed on survey	Visual (video)	3
	Hands-on	10*
	Listening (to a speaker)	1
	Reading	2
	Another way	0

Note: Respondents were allowed to answer multiple times, “Another way” was a write in answer space

* Highest number of responses

a. Table 3 is from the dot survey and shows the main question being asked “How do you learn best?”. Answers are listed with the numbers of stickers placed in their respective box,

## Data Availability

All relevant data supporting the findings of this study were included. No additional datasets beyond what is mentioned were generated in this study.
